# Recurarization in the Post-anesthesia Care Unit One Hour After Sugammadex Administration: A Case Report

**DOI:** 10.7759/cureus.108952

**Published:** 2026-05-16

**Authors:** Filipa Felix, Filipa Marques, Gabriela Tenreiro, Joana Torres, Sandra Carneiro

**Affiliations:** 1 Anesthesiology, Hospital Pedro Hispano, Matosinhos, PRT

**Keywords:** anesthesia complication, elderly, neuromuscular blockade, recurarization, rocuronium bromide

## Abstract

Postoperative recurarization has been a well-documented phenomenon since the advent of neuromuscular blockade drugs in clinical practice. Here, we present a case of recurarization after the use of rocuronium bromide in the post-anesthesia care unit (PACU), despite sugammadex administration and neuromuscular monitoring. A 68-year-old man with known psychiatric and obstructive pulmonary pathology underwent emergency surgery for abdominal evisceration correction. General anesthesia was induced with fentanyl, propofol, and rocuronium. At the end of the procedure, neuromuscular blockade was reversed with 200 mg of sugammadex under neuromuscular monitoring (train-of-four stimulation ratio >95%). The patient was transferred to the PACU 10 minutes after the end of the surgery. In the PACU, 4 mg of morphine were administered for analgesia. Fifty minutes later, peripheral oxygen saturation was 98% with a 2 L nasal cannula. The patient remained hemodynamically stable, but the Glasgow Coma Scale decreased to 3. Despite the administration of naloxone, no significant improvement in ventilation was observed. Arterial blood gas analysis revealed a high partial pressure of carbon dioxide at 82 mmHg, indicating severe respiratory insufficiency. Facial mask ventilation with a fraction of inspired oxygen of 100% was initiated. The patient recovered respiratory drive and consciousness in 15 seconds after 200 mg of sugammadex was administered. A cerebral tomography was performed to exclude central causes. After a prolonged stay in the PACU, the patient met the criteria for discharge to the ward. He died six days after surgery due to his underlying pathology and frailty.

## Introduction

Postoperative respiratory insufficiency may be due to numerous factors that need to be systematically reviewed. Recurarization is a rare possible cause and is characterized by an increase in neuromuscular blockade following a period of recovery. The current incidence of this event is reported to be 0.2% [1]. Many factors may contribute to this event, including those related to drug administration and patient factors. The incidence is notably higher in elderly patients, cases of neuromuscular relaxant overdose, and situations where monitoring is inadequate [1-3]. Diagnosis is usually made by monitoring neuromuscular blockade, and treatment options include ventilatory support and administration of sugammadex.

Here, we report a case of respiratory depression secondary to late recurarization in a patient who underwent emergency surgery under general anesthesia. The event occurred one hour after the administration of sugammadex and extubation. We highlight that recurarization should be considered a differential diagnosis for postoperative respiratory failure and emphasize the importance of performing intraoperative neuromuscular monitoring and adjusting the dose to the patient’s physical characteristics and status.

## Case presentation

A 68-year-old man weighing 50 kg underwent emergency surgery for abdominal evisceration correction. The patient had a personal history of psychiatric disorder and chronic obstructive pulmonary disease and was medicated with lithium 600 mg/day, carbidopa/levodopa 25/100 mg, quetiapine 550 mg, amlodipine 5 mg, acetylsalicylic acid 100 mg, and indacaterol/glycopyrronium bromide 110/50 mg. On the day of the surgery, his serum lithium level was 0.2 mEq/L (reference range: 0.60-1.20 mEq/L), and his serum sodium level was 143 mEq/L (reference range: 138-145 mEq/L). The patient had no reported allergies to drugs or foods. He was admitted to the emergency department 54 days earlier with a distributive shock due to Ogilvie syndrome and underwent a total colectomy with ileostomy. Immediate postoperative care occurred in the intensive care unit. The patient was discharged to the ward after 14 days of favorable evolution. During hospitalization, it was necessary to manage peritonitis with antibiotic therapy, as well as myopathy due to prolonged hospitalization. Forty-three days after the first surgery, as the patient was diagnosed with a new sub-occlusive condition refractory to conservative therapy, a surgical approach became necessary. The postoperative period was complicated by pneumonia. A third surgery was performed seven days after the second surgery to correct an evisceration.

Fifty-four days after admission, new evisceration was documented and the patient underwent the fourth surgical procedure. In the operating room, he was monitored with American Society of Anesthesiologists standard monitoring, urinary output, processed electroencephalogram, and neuromuscular blockade. Monitoring of neuromuscular blockade was performed with an acceleromyography monitor placed inside the left forearm. Prior supramaximal train-of-four (TOF) stimulation (>50 mA) calibration was performed and a train-of-four ratio (TOFR) was applied every 15 minutes. TOFR values were displayed on the monitor screen during the entire procedure.

The patient was preoxygenated via a face mask with oxygen (10 L/ minute) for three minutes. Intravenous induction of general anesthesia was achieved with 100 µg of fentanyl (2 µg/kg), 50 mg of lidocaine (1 mg/kg), and 60 mg of propofol (1.2 mg/kg). After loss of consciousness, 100 mg of rocuronium (2 mg/kg) was administered, and oral intubation was successfully performed using a 7.5 endotracheal tube. General anesthesia was maintained with an air-oxygen mixture, sevoflurane, and intermittent doses of fentanyl (50 µg). No supplemental rocuronium was administered after the initial dose used for tracheal intubation. Given the frailty of the patient and a history of hemodynamic instability during previous surgeries, an infusion of noradrenaline was maintained via central venous access, which allowed hemodynamic stability during the entire procedure, and it was suspended at the end of the surgery.

**Figure 1:**
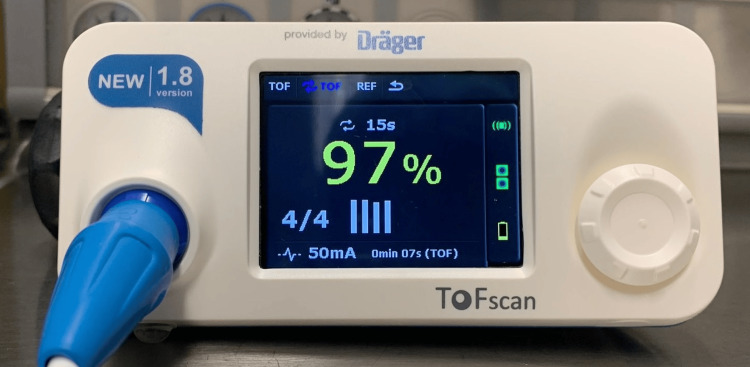
Monitoring of neuromuscular blockade before extubation.

Surgery was completed in 58 minutes without complications. Paracetamol 1,000 mg and tramadol 50 mg were given intravenously for analgesia. At the end of the surgery, 64 minutes after the administration of rocuronium, no muscle contraction of the adductor pollicis occurred with TOF stimulation. Post-tetanic count (PTC) was performed to decide sugammadex dosage. Neuromuscular blockade monitor showed a PTC value of 17 and 200 mg of sugammadex was administered. Total reversal of neuromuscular blockade was confirmed by TOFR >95% (Figure 1). Extubation was performed with the patient in spontaneous ventilation, exhibiting an adequate respiratory rate and tidal volume, with open eyes and responsiveness to simple commands.

Ten minutes after extubation, the patient was transferred under clinical monitoring to the post-anesthesia care unit (PACU). On arrival at the recovery room, oxygen saturation was 98%, heart rate was 124 beats/minute, and mean arterial pressure was 96 mmHg. During the recovery period, the patient was always monitored with electrocardiogram, pulse oximetry, and non-invasive blood pressure. A total of 4 mg of morphine was administered for pain control.

Fifty minutes after admission to the PACU, despite hemodynamic stability, the medical team verified that the patient was unresponsive to painful stimuli (Glasgow Coma Scale score of 3) and had a low respiratory rate. An arterial blood gas sample was collected and revealed a PCO_2_ of 82 mmHg and oxygen saturation of 100%. A total of 4 mg of naloxone was administered, followed by a further 0.4 mg to reverse a possible opioid-induced respiratory depression, with no response. Due to progressive respiratory depression, face mask ventilation (FiO_2_ 100%) was initiated and 200 mg of sugammadex was administered. At the time, neuromuscular blockade was not monitored, but 15 seconds after sugammadex administration, the patient began to recover respiratory drive and consciousness. No clinical signs of residual paralysis were observed.

To exclude a central cause of the event, a cerebral image was obtained, and no relevant findings, such as ischemia or hemorrhage, were noted. After two hours of monitoring in the PACU, the patient met the discharge criteria to the ward, as he had fully regained consciousness and showed no deficits. However, given the patient’s frailty and the severity of the underlying pathology, the patient died on postoperative day six.

## Discussion

This case presents an episode of altered consciousness caused by narcosis due to late recurarization following the reversal of neuromuscular blockade with sugammadex. Recurarization has been well-documented since the use of neuromuscular blockers in anesthesia practice. This phenomenon was more frequent with older neuromuscular blockers such as pancuronium and d-tubocurarine [1,2]. Nowadays, with the most recent drugs, the awareness to monitor neuromuscular block, and the use of neuromuscular block antagonists, the prevalence of this phenomenon has reduced from 42% to 0.2% [2]. Although recurarization was not confirmed with a neuromuscular blockade monitor, some clinical factors point to the likely diagnosis of late recurarization. The absence of residual opioid effect after naloxone administration, recovery of respiratory drive seconds after sugammadex administration, and the fact that a high dose of rocuronium was administered during the induction period are some of the clinical factors that support our diagnosis.

During the induction period, 100 mg of rocuronium bromide (2 mg/kg) was administered. Rocuronium is an intermediate-acting steroidal neuromuscular blocking agent (NMBA), which is soluble in water and highly ionized and is frequently used to facilitate tracheal intubation [3]. Rocuronium binds, at the neuromuscular junction (NMJ), to the acetylcholine (ACh) receptors in a competitive inhibitory manner with ACh. It occupies the majority of the postsynaptic ACh receptors, preventing the influx of sodium ions and consequently inhibiting end-plate depolarization and causing skeletal muscle paralysis [3].

As we decide to perform a rapid sequence induction, rocuronium doses should be formally determined based on the patient’s weight (1.2 mg/kg) to facilitate intubation conditions and reduce the risk of regurgitation. Dosing recommendations for non-depolarizing NMBAs are generally based on the patient’s total body weight (TBW). However, the pharmacology of NMBAs differs in patients according to body composition (quantity of skeletal muscle). For individuals with a limited amount of skeletal muscle, TBW-based dosage may prolong the duration of action of NMBAs [3].

Sarcopenia is characterized by a progressive loss of muscle mass and function. The reduction of muscle fibers, including the reduction in the number and function of motor neurons, reduction and critical disruption of mitochondria with an impact on energy production and transduction signal regulation, decreased synaptic vesicles and enzyme function, and progressive uncoupling of the excitation-contraction mechanism are some characteristics that justify the impact of sarcopenia on the pharmacokinetics of NMBAs [4]. Muscular activity increases the number of ACh receptors at the NMJ through neuronal and trophic factors [4].

Patient safety can be enhanced through continuous monitoring and routine reversal of neuromuscular blockade. In this instance, the patient was monitored using acceleromyography, and 200 mg of sugammadex was administered. To define the dose of sugammadex, the weight and depth of the neuromuscular blockade should be considered. A deep blockade (TOF <2) should be reversed with 4 mg/kg, and in the case of TOF >2, the recommended dose is 2 mg/kg [1].

**Figure 2:**
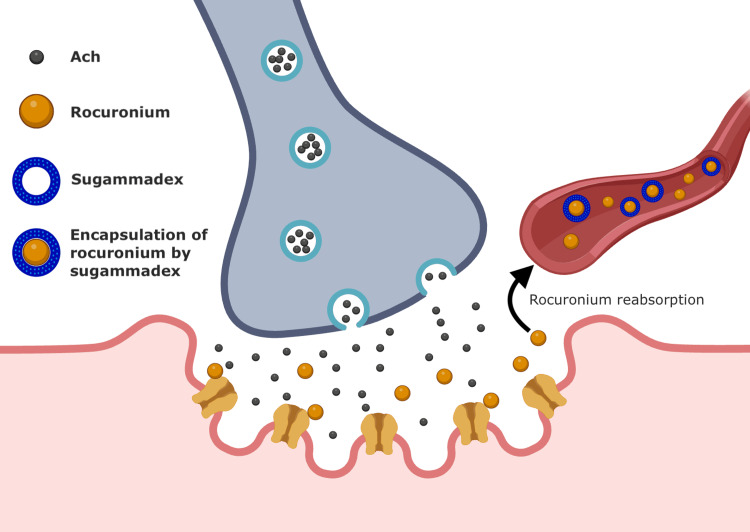
Reabsorption of rocuronium from the neuromuscular junction and encapsulation of free rocuronium in plasma by sugammadex. Adapted from Boon et al. 2018 [6].

Sugammadex has a high affinity to rocuronium; one molecule of sugammadex binds to one molecule of rocuronium and unbinding is very unlikely. However, their distribution by compartments is distinct because sugammadex has a low volume of distribution and is almost restricted to intravascular space [1]. Therefore, because the more prolonged duration of action of rocuronium depends mainly on redistribution to deep compartments, sugammadex may not be sufficient to completely reverse rocuronium overdose in the third space (Figure 2) [3]. The first dose of sugammadex administered at the end of surgery binds to rocuronium molecules present in the intravascular space and at neuromuscular junctions, thus restoring the neuromuscular function. Due to the subsequent reduction of rocuronium concentration in these spaces, there is a shift from peripheral components to the intravascular space. In the case of inadequate low doses of sugammadex, all molecules of sugammadex are already occupied allowing the remaining rocuronium molecules to bind again to ACh receptors, leading to recurarization. In this case, although 4 mg/kg of sugammadex had been administered, it seems to have been insufficient. We also believe that, given the reduced muscular mass, the accuracy of neuromuscular block monitoring may not have been ideal, contributing to the inadequate selection of sugammadex dose [5].

In cases of suspected late recurarization, neuromuscular monitoring should be used. Although immediately available in the unit, sugammadex was administered based solely on clinical factors due to the emergency.

Another factor that might have contributed to late recurarization was the presence of lithium in the blood [7]. Several mental disorders such as bipolar affective disorder, mania, recurrent depression, and self-mutilating behavior are treated with lithium carbonate. It is a monovalent cation from the alkali metal group, and it is not metabolized, being mostly excreted by the kidneys. While the precise pharmacological impact of lithium is unknown, it has various psychiatric effects, including the reduction of noradrenergic adenylate cyclase activity and the enhancement of presynaptic noradrenaline reuptake [7]. As the size of the lithium ion is comparable to that of sodium, it is transported into the cell with sodium. The resting membrane potential declines and consequently the action potential height. As a result, the rocuronium-induced neuromuscular blockade may have been somewhat offset by the action of the remaining lithium. According to reports, both depolarizing and non-depolarizing muscle relaxants are potentiated by lithium [7]. However, given the confirmation of subtherapeutic levels, we believe that lithium had little significance in this case.

## Conclusions

This clinical case represents a rare complication of late recurarization with rocuronium in the PACU, despite neuromuscular monitoring and sugammadex administration. Subsequently, upon reflection, we determined that the most likely cause of this incident was an overdose of rocuronium, given the patient’s significant sarcopenia and prolonged immobilization.

In addition to TBW, the body composition of frail patients should be considered when dosing NMBAs, and neuromuscular blockade should always be monitored during reversal.
